# Proton conductivity of glycosaminoglycans

**DOI:** 10.1371/journal.pone.0202713

**Published:** 2019-03-08

**Authors:** John Selberg, Manping Jia, Marco Rolandi

**Affiliations:** Department of Electrical Engineering, University of California, Santa Cruz, CA, United States of America; US Naval Research Laboratory, UNITED STATES

## Abstract

Proton conductivity is important in many natural phenomena including oxidative phosphorylation in mitochondria and archaea, uncoupling membrane potentials by the antibiotic Gramicidin, and proton actuated bioluminescence in dinoflagellate. In all of these phenomena, the conduction of protons occurs along chains of hydrogen bonds between water and hydrophilic residues. These chains of hydrogen bonds are also present in many hydrated biopolymers and macromolecule including collagen, keratin, chitosan, and various proteins such as reflectin. All of these materials are also proton conductors. Recently, our group has discovered that the jelly found in the Ampullae of Lorenzini- shark’s electro-sensing organs- is the highest naturally occurring proton conducting substance. The jelly has a complex composition, but we proposed that the conductivity is due to the glycosaminoglycan keratan sulfate (KS). Here we measure the proton conductivity of hydrated keratan sulfate purified from Bovine Cornea. PdH_x_ contacts at 0.50 ± 0.11 mS cm ^-1^, which is consistent to that of Ampullae of Lorenzini jelly at 2 ± 1 mS cm ^-1^. Proton conductivity, albeit with lower values, is also shared by other glycosaminoglycans with similar chemical structures including dermatan sulfate, chondroitin sulfate A, heparan sulfate, and hyaluronic acid. This observation supports the relationship between proton conductivity and the chemical structure of biopolymers.

## Introduction

**Fig 1:**
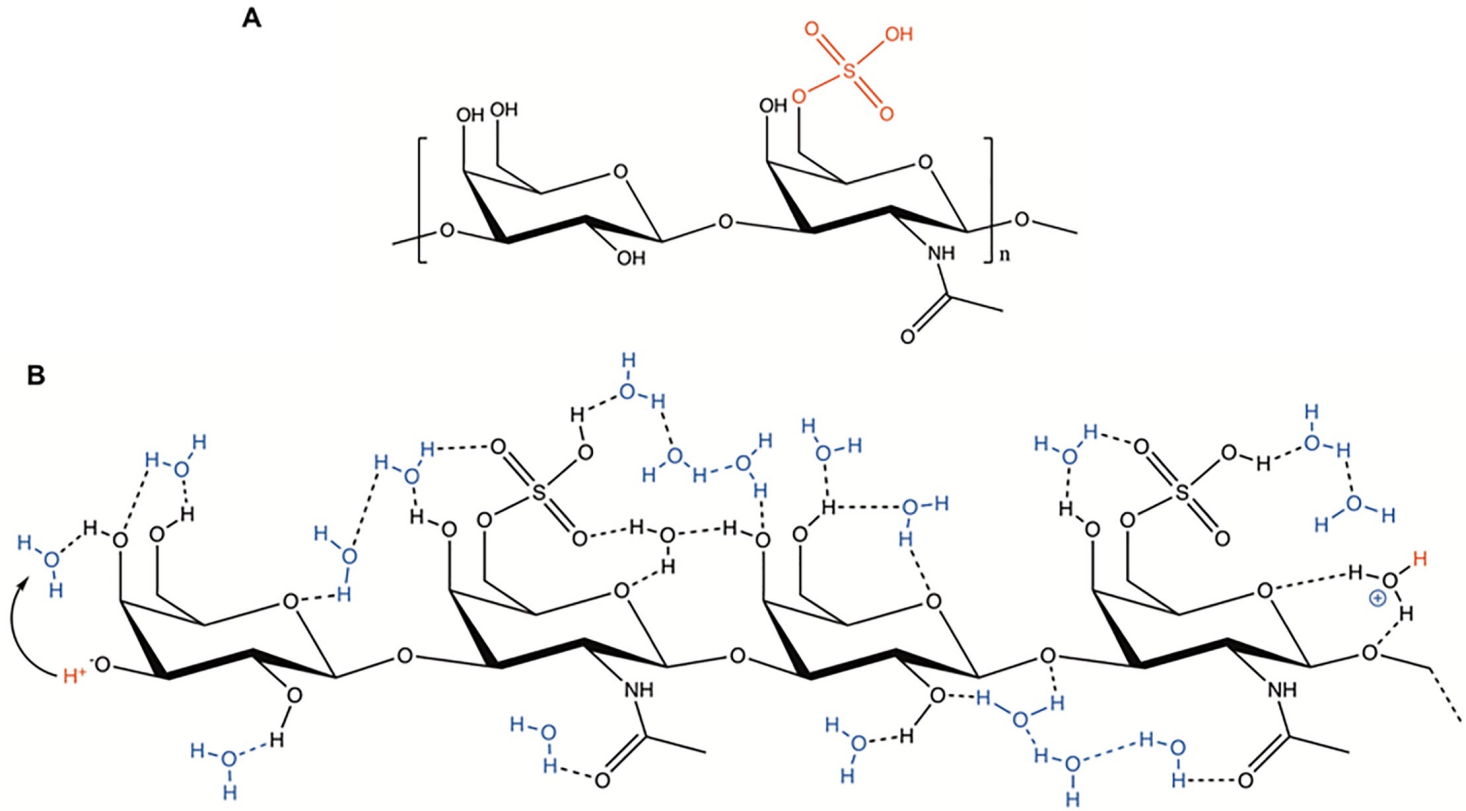
The keratan sulfate. **(A)** Chemical structure of KS. **(B)** An illustration of a three-monomer segment of KS. Possible intra- and inter-molecular hydrogen bonds as well as the hydrogen bonds between the water of hydration and the polar parts of the molecule form a continuous network comprised by hydrogen-bond chains. The sulfate group interacts with the hydrogen-bond network and forms an H_3_O^+^ (hydronium) ion.

Proton (H^+^) conductivity is important in many natural phenomena[1] including oxidative phosphorylation in mitochondria and archaea[2–4], uncoupling membrane potentials by the antibiotic Gramicidin[5], and proton actuated bioluminescence in dinoflagellate[6]. In all of these phenomena, the conduction of H^+^ occurs along chains of hydrogen bonds between water and hydrophilic residues. These chains are often referred to as proton wires[3]. This conduction follows the Grotthus mechanism in which a hydrogen bond is exchanged with a covalent bond contributing to the effective transfer of an H^+^ from a molecule to its next-door neighbor[7]. Following this mechanism, proton conductivity in hydrated biopolymers and macromolecules is widespread including collagen[8], keratin[9], chitosan[10], melanin[11], peptides[12], and various proteins such as bovine serum albumin[13] and reflectin[14, 15]. In addition to the ability to support proton wires, typically these materials include an acid or a base group that serve as H^+^ or OH^-^ dopants and provide charge carriers for proton conductivity [16–18]. Following this trend, for example, the synthetic polymer Nafion, with a high proton conductivity of 78 mS cm^-1^, contains very strong acid groups that donate H^+^ to the water of hydration for proton conduction [19]. Our group has recently demonstrated that the jelly contained in the ampullae of Lorenzini, the electrosensing organ of sharks and skates, is the highest naturally occurring proton conductor[20]. We proposed that keratan sulfate (KS), a glycosaminoglycan (GAG), was the material responsible for proton conductivity due to its similar chemical structure to other known proton conductors such as chitosan, and the ability to form many hydrogen bonds with water when hydrated (Fig 1A)[21, 22]. Given that it is difficult to purify KS from the shark jelly due to small amounts of sample per organism, we set to explore KS from different sources that were available to perform these measurements. Here, we have measured the proton conductivity of KS derived from bovine cornea [23, 24] and other GAGs using Pd based proton conducting devices [10].

GAGs are long, linear, hydrophilic biopolymers composed of repeating of disaccharide units with many acidic groups that may support the presence of proton wires (Fig 1B) that transport protons through the Grotthuss mechanism [25]. Among these are hyaluronic acid (HA), heparan sulfate (HS), chondroitin sulfate A (CSA), dermatan sulfate (DS), and KS[26, 27]. Additionally, GAGs have important biological functions in regulating hydration and water homeostasis of tissues, which is derived from their ability to absorb very large amounts of water at high humidity[28]. They are also implicated in many fundamental operations such as cell patterning [29], cell signaling, and regulation[30].

## Materials and methods

### Materials

Glycosaminoglycan samples were received from the Linhardt laboratory at Rensselaer University and stored dry at -15C. Including, 70% pure CSA isolated from bovine trachea (average MW: 20kDa), HA sodium salt from streptococcus zooepidemicus (average MW: 100kDa), DS from porcine intestinal mucosa (average MW: 30kDa), HS (porcine intestinal mucosa (average MW: 14.8kDa), and KS isolated from the bovine cornea (average MW: 14.3kDa) a biochemical description of the KS can be found at Weyers et al.[24].

### Device fabrication

Two-terminal measurements were performed on Si substrates with a 0.1μm SiO_2_ layer. Conventional photolithography was used to pattern 0.1μm thick Au and Pd contacts. Pd contacts were 500 μm wide and separated by different channel lengths, L_SD_ = 5, 10, 20, 50, 100, 200, 500 μm. We performed both two terminal device measurements and transmission line measurements (TLM) to reduce the influence of contact resistance on the conductivity [11].

### Deposition of glycosaminoglycans

All lyophilized samples were rehydrated in DI water (pH 6.7) at a concentration of 0.15–0.2 mg μl^-1^ and drop casted onto the devices. The samples were the dehydrated into a film with dry nitrogen gas flow.

### Proton conductivity measurements

Direct current–resistance measurements were performed using a Keithley 4200 source-meter and a two-contact probe station arrangement on devices. The devices were enclosed in an environmental chamber at room temperature in an atmosphere of nitrogen or hydrogen with controlled relative humidity (RH). We controlled RH by bubbling gases through a bubbler containing DI water at pH 6.7. Hydrated in sequence from dry to 75%RH in N_2_, 90%RH in N_2_, 90% RH in a mixture of 95% N_2_ with 5% hydrogen, and 90% RH in a mixture of 95% N_2_ with 5% deuterium gas to form PdH_x_ or PdD_x_ contacts. A one-hour incubation period was carried out after switching between humidity and gas compositions. During the measurement, the Pd/PdH_x_ electrodes were contacted with tungsten probes. When we applied a source-drain potential difference, V_SD_, the PdH_x_ source injected protons (H^+^) into drain through the samples, inducing measurable electrical current in the circuit.

## Results and discussion

### Proton conductivity measurements

Palladium (Pd) devices are useful for studying proton transport in materials due to the nature of Pd to reversibly form palladium hydride (PdH_x_)[31–34]. Several mechanisms for the formation of PdH_x_ are known (Eqs 1–4).

$$H_{2}+Pd\to 2PdH_{ads}$$ (1)

Equation one describes the adsorption and splitting of H_2_ molecules into two adsorbed H on the Pd metal surface without electron transfer in a reaction described by Tafel kinetics.

$$H_{2}+Pd\to PdH_{ads}+H^{+}+e^{-}$$ (2)

Equation two is the Heyrovsky reaction in which a H_2_ is split into an adsorbed H atom and a H^+^, e^-^ pair at the Pd surface, this e^-^ is transferred into the metal.

$$H^{+}+Pd+e^{-}↔PdH_{ads}$$ (3)

The Volmer reaction in Eq 3 describes a third mechanism, which involves an electron transfer to a H^+^ near the Pd surface allowing it to adsorb as PdH_ads_. Once PdH_ads_ is formed on the metal surface, H can diffuse into the subsurface bulk forming PdH_x_ (Eq 4). [10, 35, 36].

$$PdH_{ads}↔PdH_{x}$$ (4)

**Fig 2:**
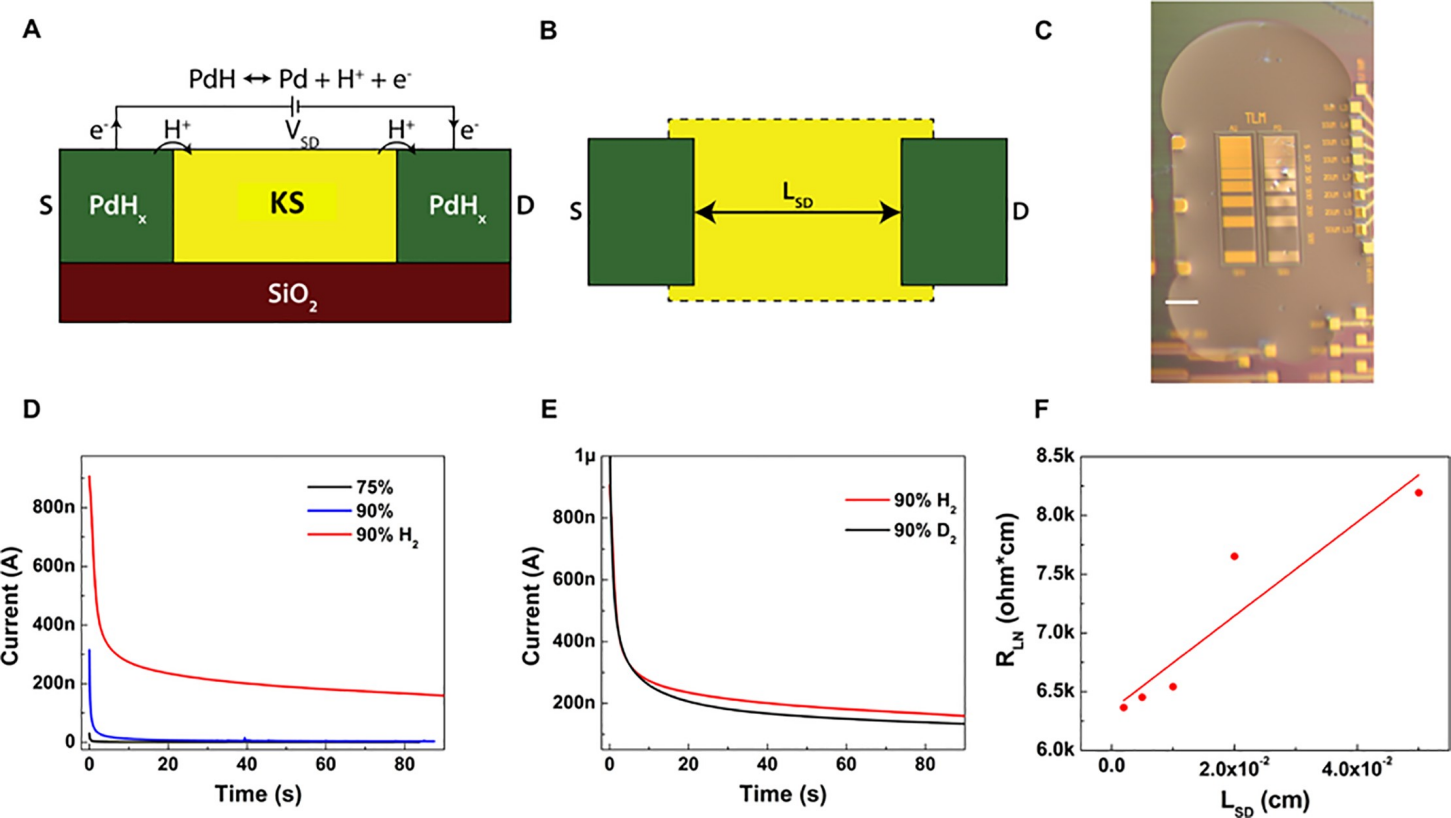
Proton conduction measurement of KS. **A)** Palladium hydride(PdH_x_) electrode behavior. Under a V_SD_, PdH_x_ source split into Pd, H^+^, and e^−^. Protons are injected into the KS, whereas electrons travel through external circuitry and are measured. **B)** TLM geometry. Varying the distance between source and drain (L_SD_) distinguishes between the fixed PdH_x_−KS interface contact resistance and the varying bulk resistance. **C)** Optical image of TLM geometry with hydrated KS on the surface. Scale bar, 500μm. **D)** Transient response to a 1V bias in KS at 75%, 90%, 90% H_2_ RH, in which the current under 90% with hydrogen is much higher than that under 90% RH without hydrogen. **E)** Deuterium current (black) at 90% D_2_ humidity is lower than proton current (red). **F)** The normalized resistance R_LN_ as a function of L_SD_, A linear fit gives a bulk material proton conductivity of 0.50 ± 0.11 mS cm^-1^.

Pd devices were designed such that PdH_x_ formation occurs spontaneously by Eq 1 in a 5% H_2_ atmosphere on two Pd contacts. These Pd/PdH_x_ contacts are separated by a channel consisting of a GAG film which completes the circuit (Fig 2A and 2B). A voltage V_SD_ between the Pd/PdH_x_ contacts induces a current of H^+^ to exit one Pd contact, travel through the film channel, and enter the second Pd contact according to Eq 3. In this manner, one e^-^ travels through the circuit and is recorded as I_d_ for each H^+^ that is conducted through the channel. Considering the conductivity of the GAG films is expected to be much less than the conductivity of electrons in electrodes, the current that we measure during the experiments is indicative of the conductivity of the channel.

### Materials characteristics

After deposited directly onto the transmission line measurement (TLM) (Fig 2C) device surface without further processing, the KS film is thick, viscous, and optically transparent. After one hour of incubating at 50%RH, the KS film dries to a non-homogenous film. The film rehydrates fully after incubating at 90%RH for one hour and appears as wet as when it was drop-cast form solution (Fig 2C). This high water content of KS films is a result of sulfate groups functionalizing either or both of the galactose and N-acetyl glucosamine sugars which make up the repeating disaccharide unit of the GAGs. Considering the other members of GAGs family, DS, HS, CSA, and HA also contain an abundance of repeating acidic groups which may stabilize proton wires, as shown in Table in S1 File.

### DC electrical measurements with PdH_x_ proton-conducting contacts

With V_SD_ = 1V on the Pd devices, we measured the drain current (I_D_) of KS, as shown in Fig 2D. First, at 75% RH in N_2_, I_D_ (~ 0.5 nA) is small (black in Fig 2D). With the RH increased to 90% in N_2_, the increase in I_D_ was negligible (red in Fig 2D). However, after we changed the gas to 95%N_2_ + 5%H_2_, the I_D_ increased more than 300 times to 155 nA (green in Fig 2D). The same measurements were performed with DS, HS, CSA, and HA family and followed similar trends (Figure A in S1 File). All GAGs displayed an increased current upon a 90%RH (5%H_2_) atmosphere compared to a 90%RH N_2_ atmosphere, indicating that protons predominately contribute to the conductivity of GAGs materials at high relative humidity.

### Kinetic isotope effect

To further test whether KS conductivity predominantly arises from protons, we investigated the kinetic isotope effect. Measurements were repeated while hydrating the sample with deuterium oxide (D_2_O) instead of water and exposing the sample to deuterium gas rather than H_2_. Like protons, deuterium ions (D^+^) can transport along proton wires and hydrated materials, albeit with a lower mobility and an associated lower current due to the higher molecular weight and higher binding energy during H-bonding[37]. The kinetic isotope effect in KS is evident as a drop in the conductivity when deuterium replaces hydrogen as the atom being transported (Fig 2E). Here, we observe a 15% drop in current when deuterium replaces hydrogen. The kinetic isotope effect observed with KS is relatively small. However, a similar small kinetic isotope effect was observed for the proton conduction of bovine serum albumin[13]. The other members in GAGs family display a larger kinetic isotope effect, the current drop is nearly 50% (Figure B in S1 File). The divergence of the KIE between the KS films and the other GAGs may be due to regions different transport regimes for H^+^ in KS films. Where the binding energy plays a big role in H-bond mediated transport by the Grotthuss mechanism it will not be as noticeable by regions of bulk diffusion.

### Transmission line measurement

TLM devices are designed with different lengths between the Pd source and the drain contacts to eliminate the effect of contact resistance in the measurements of the proton conductivity (Fig 2B) [20]. We applied V_SD_ = 1 V on devices with L_SD_ ranging from 5 to 500 um, measured I_D_, and calculated the resistance of each device, R_L_. In this geometry, R_L_ increases linearly with L_SD_, but the contact resistance, R_C_, at the source–KS and drain–KS interface is constant. Considering that different devices contained KS with different thicknesses, we multiplied R_L_ by the sample thickness to get the normalized resistance, R_LN_. The slope of the plot of R_LN_ as a function of L_SD_ is proportional to the resistivity of KS, and the intercept on the R_LN_ axis for L_SD_ = 0 is R_CN_ (Fig 2F). Here, we obtain σ = 0.50 ± 0.11 mS cm^-1^, which is only one order of magnitude lower than the proton conductivity of Nafion σ = 58.3 ± 2.5 mS cm^-1^[20] measured in the same geometry (Figure C in S1 File). The proton conductivity of the Nafion control sample (58.3 ± 2.5 mS cm^-1^) measured in a TLM geometry is extremely close to the reported value of 78 mS cm^-1^.[19] Therefore, we conclude that σ = 0.50 ± 0.11 mS cm^-1^ measured in this way is a good indicator of the proton conductivity of KS. Table 1 shows the proton conductivity of Nafion and known biopolymers, and KS performs well among them.

**Table 1:** Room-temperature proton conductivities of Nafion and known biopolymers.

Materials	Conductivity (mS cm^-1^)	Ref
Nafion	78	[19]
AoL jelly	2 ± 1	[20]
Keratan Sulfate	0.50 ± 0.11	This work
Maleic Chitosan	0.7	[38]
Reflectin	0.1	[14]
Bovine Serum Albumin	0.05	[13]
Melanin	0.02	[11]

Out of the other GAG films measured and reported in Table in S1 File. Hyularonic acid has the highest conductivity 0.28 ± 0.06 mS cm^-1^. However, some of the other GAGs materials, such as dermatan sulfate, did not form a homogeneous film and it was not possible to measure the conductivity using the TLM geometry. The conductivity reported with the two terminal geometry also contains contact resistance and therefore it is lower as expected. Within experimental error, we did not observe any variation in conductivity with variation in pK_a_ of the acidic groups. It is difficult to relate the concentration of H^+^ in these hydrated states because pK_a_ is determined in infinite dilution. We assume that we are hydrating the films with water at neutral pH, then we expect the vast majority of the sulfonate acidic groups on the GAGs to become ionized independent of their individual variation in pK_a_.

## Conclusions

Inspired by the high conductivity in the jelly of the ampullae of Lorenzini, we measured the proton conductivity of KS and other glycosaminoglicans with similar chemical structures. Using TLM devices at room temperature, we measured the proton conductivities of 0.50 ± 0.11 mS cm^-1^ at 90% RH (5%H_2_)for KS, which is near to that of ampullae of Lorenzini jelly (2 ± 1 mS cm^-1^). This result supports the claim that KS is a factor in the high proton conductivity of the ampullae of Lorenzini jelly. We leave open the possibility that other materials in the ampullae of Lorenzini jelly and organization of the KS chains may play additional roles well. We have also measured the proton conductivity of other GAGs including HS, DS, CSA and HA. Their conductivity is lower, but comparable with KS suggesting that proton conductivity is a common property of GAGs with acidic groups upon hydration. In the future, chemical modification of GAGs with precise patterns of acidic groups may provide further insights in this conjecture.

## Supporting information

S1 File Supporting information. Includes S1A Fig, S1B Fig, S1C Fig, S1A Table.
(DOCX)
