# Supplementary material for: Proton conductivity of glycosaminoglycans
Source: PLoS One. 2019 Mar 8;14(3):e0202713. doi: 10.1371/journal.pone.0202713 (PMC6407855; doi:10.1371/journal.pone.0202713)
Supplement: S1 File — Includes S1A Fig, S1B Fig, S1C Fig, S1A Table. (DOCX) [file pone.0202713.s001.docx]

Proton Conductivity of Glycosaminoglycans (Supplementary Information)

**
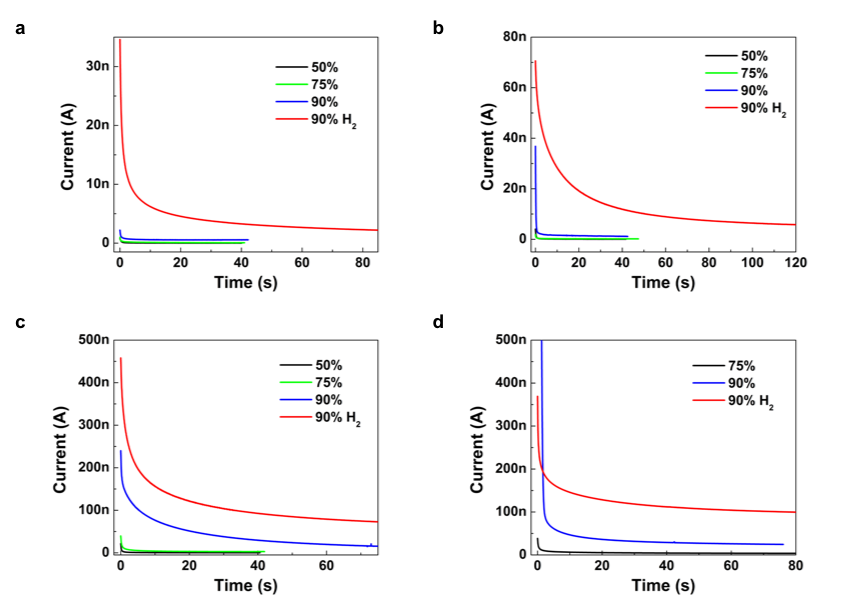
**

**S1** **Fig A.** Current under different RH of GAGs family: **(a)** hyaluronic acid, **(b)** heparan sulfate, **(c)** chondroitin sulfate A and **(d)** dermatan sulfate. The current under 90%RH with hydrogen is much higher than 90%RH without hydrogen.


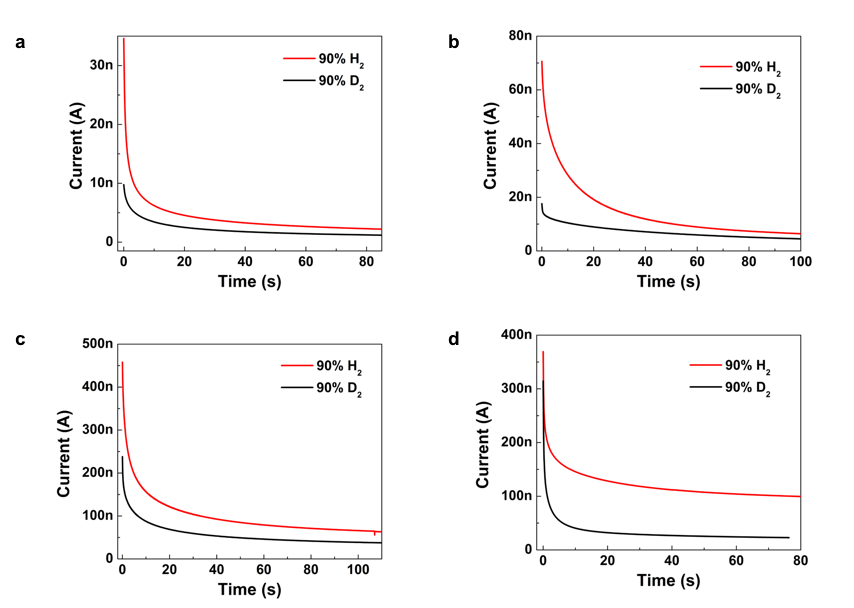


**S1** **Fig B.** Kinetic isotope effect in members of GAGs family: **(a)** hyaluronic acid, **(b)** heparan sulfate, **(c)** chondroitin sulfate A and **(d)** dermatan sulfate. Current measured in a 5% deuterium (black) atmosphere at 90%RH vs a 5% proton atmosphere at 90%RH (red).


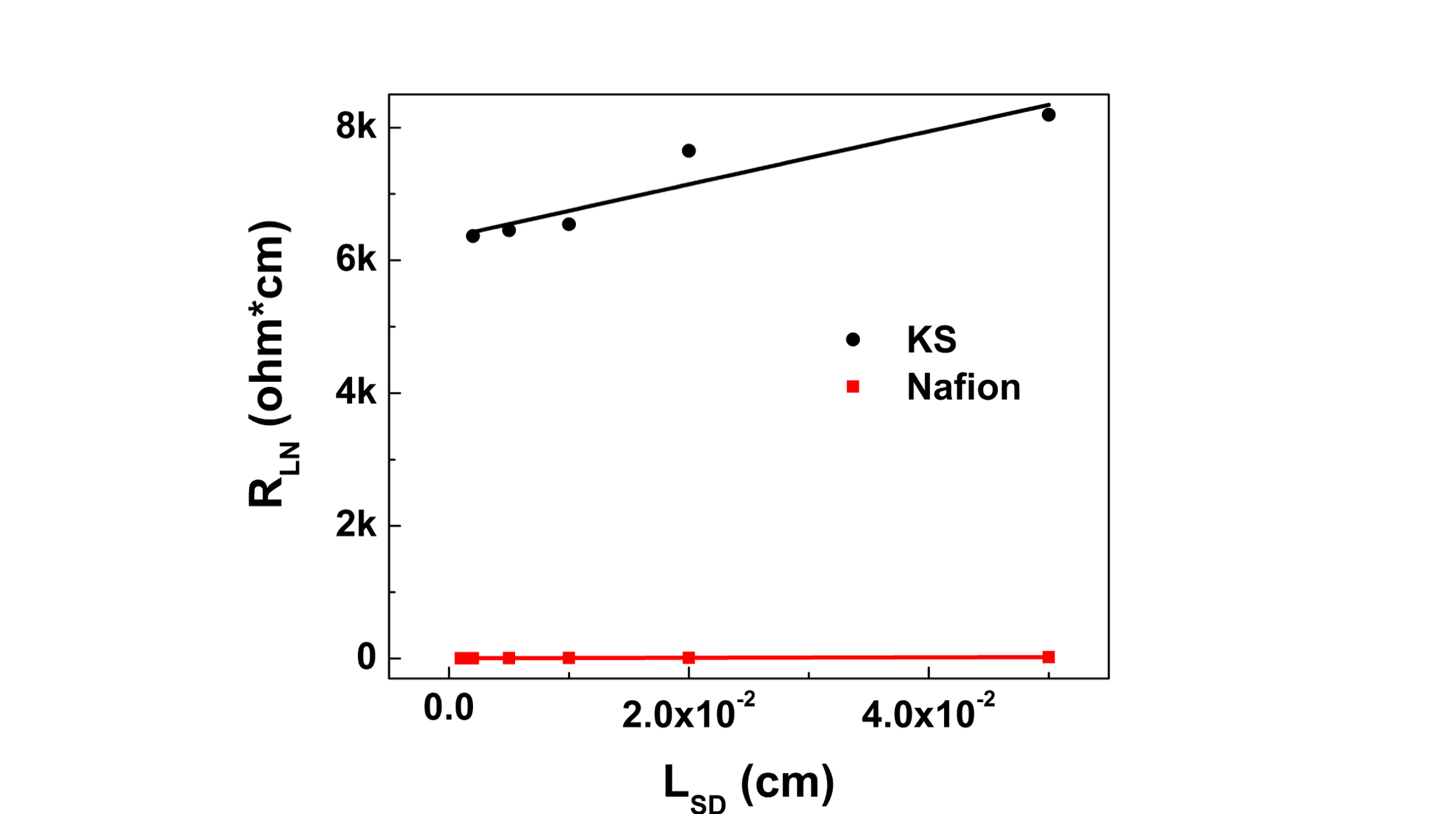


**S1** **Fig C.** Control experiments on Nafion. Conductivity of Nafion measured with this TLM device is 58.3 ± 2.5 mS cm^-1^. It’s slightly lower than the literature value of 78 mS cm^-1^, which is attributed to sample preparation. The reported literature value is after immersion in heated sulfuric acid, while the sample here was simply drop-cast from solution.

**S1** **Table** Glycosaminoglycan chemical structures, Pka and conductivity (σ) estimated with TLM devices

| Materials | Chemical structure | Pka | σ (mS cm^-1^) |  | σTLM (mS cm^-1^) |
| --- | --- | --- | --- | --- | --- |
| Keratan Sulfate | 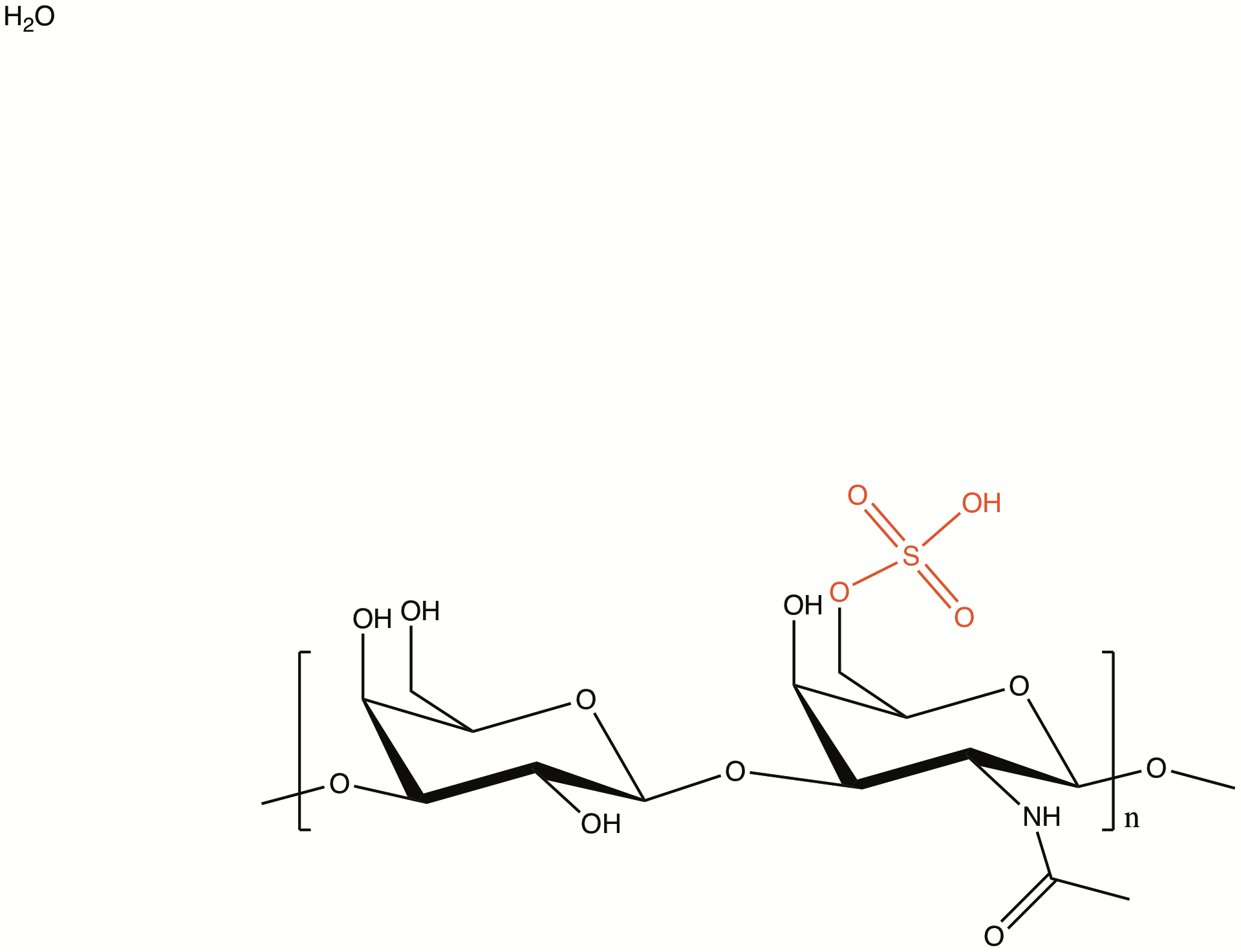 | 2[1] | 0.015 |  | 0.50 ± 0.11 |
| Dermatan Sulfate | 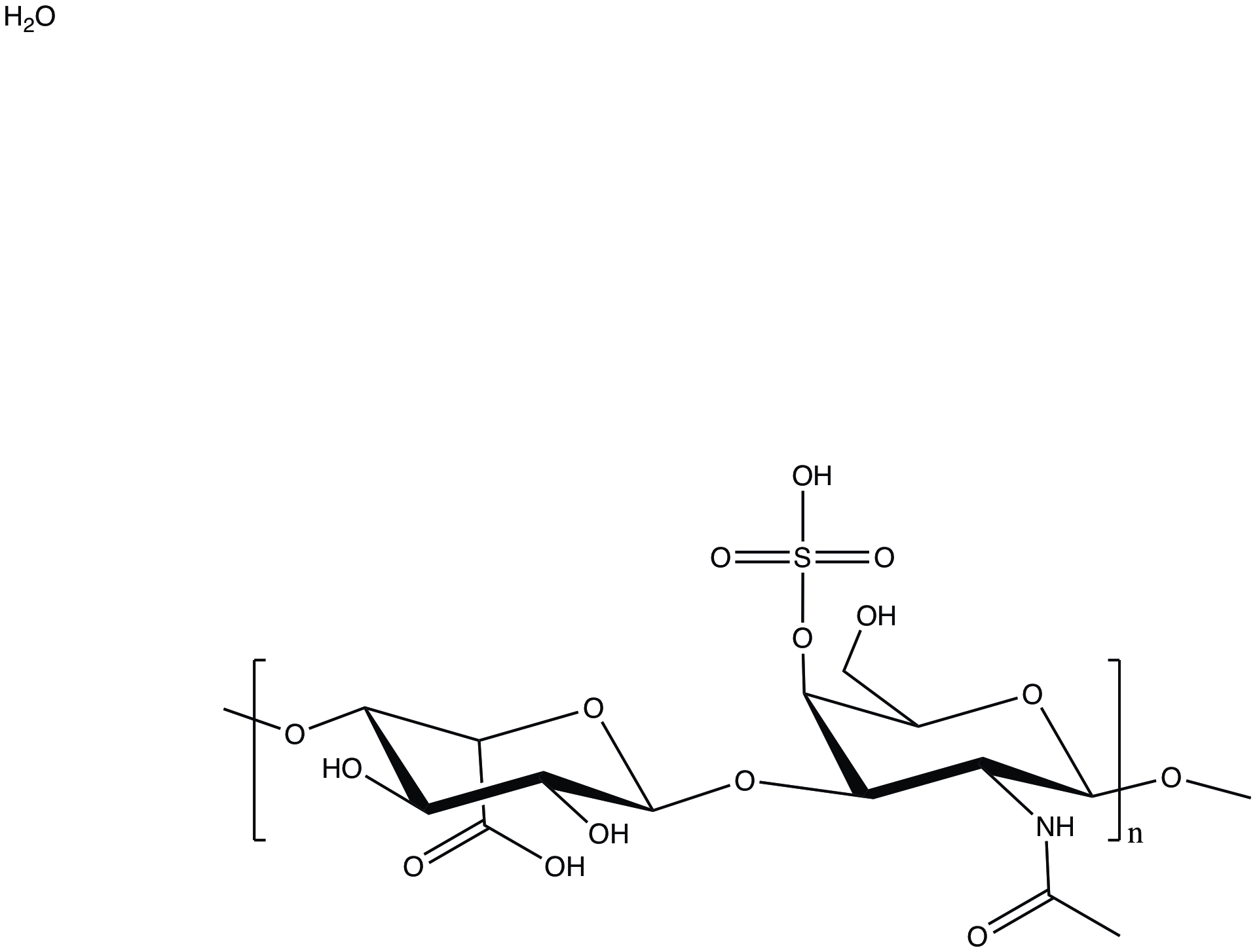 | 1.9[2] | 0.030 |  | - |
| Heparan Sulfate | 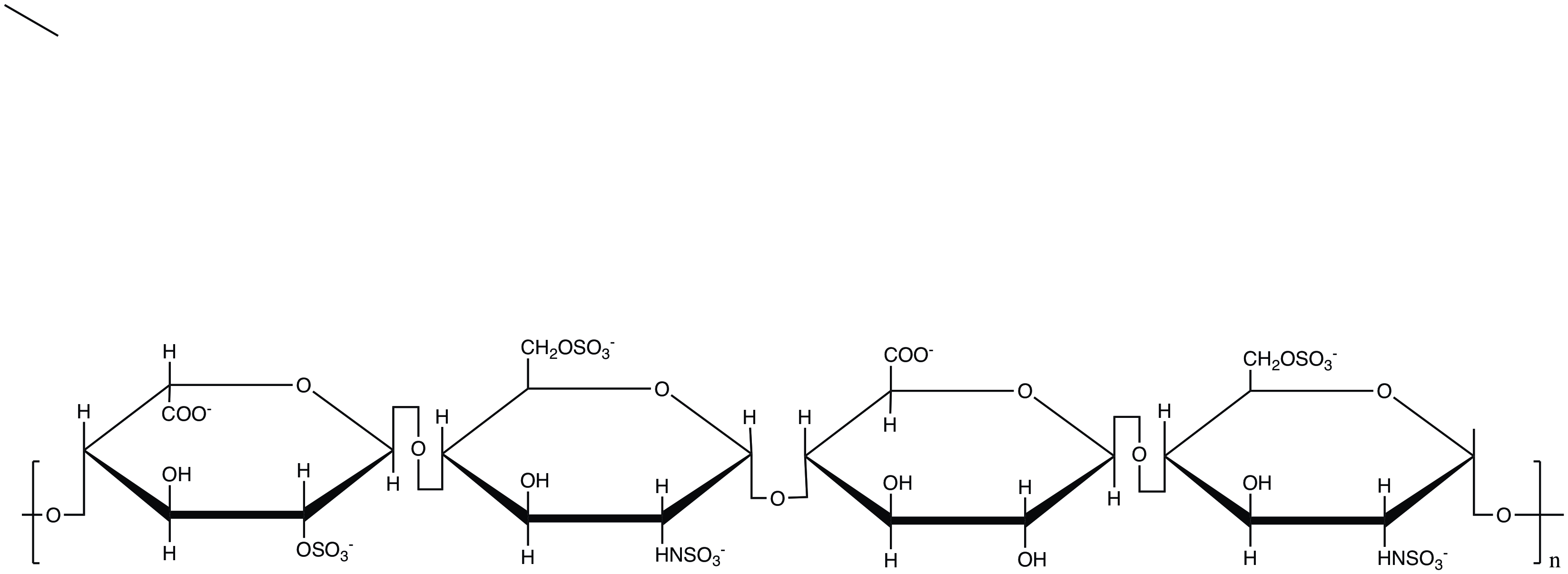 | - | 0.012 |  | - |
| Hyaluronic Acid | 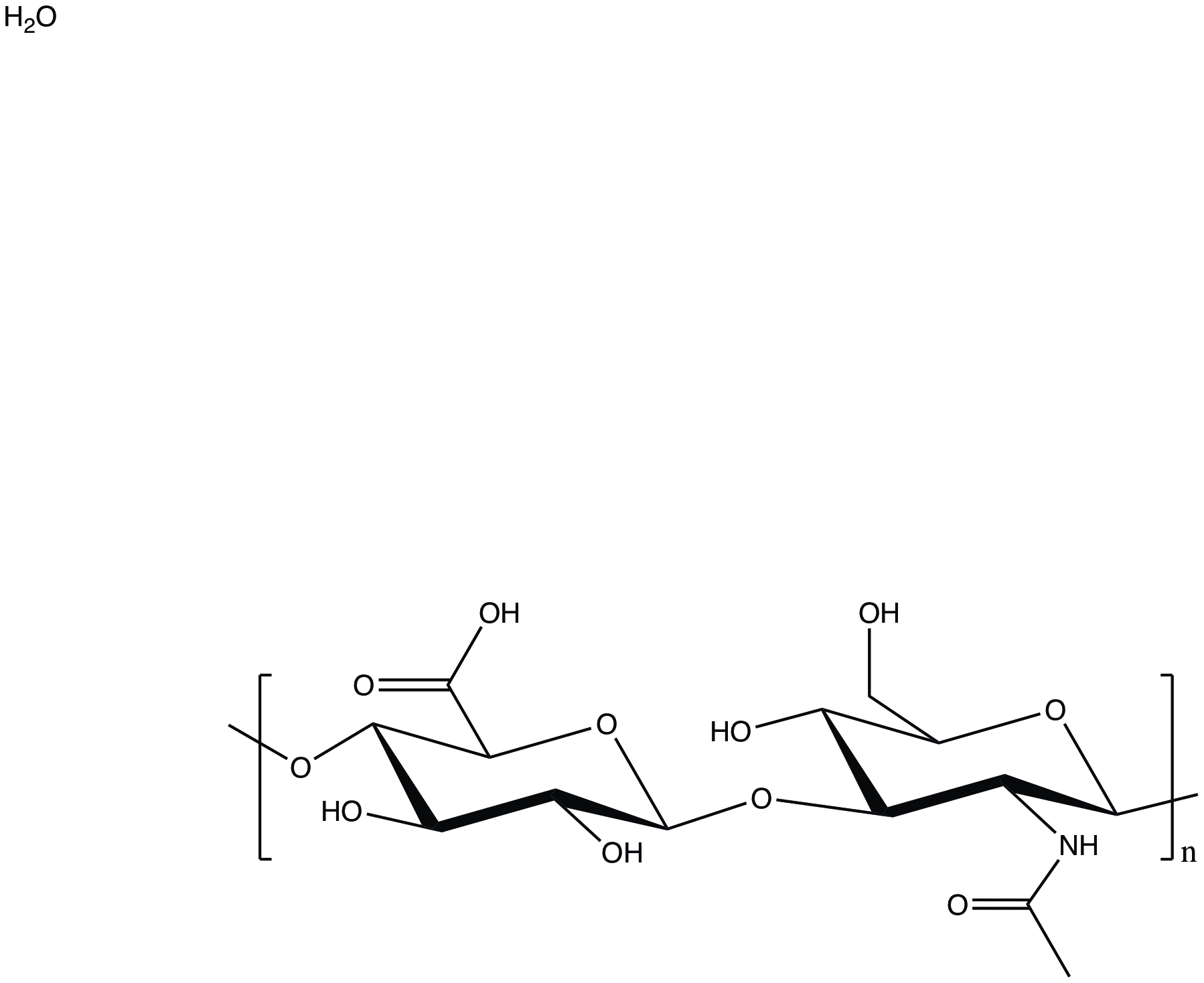 | 3.0[3] | 0.012 |  | 0.28 ± 0.06 |
| Chondroitin Sulfate A | 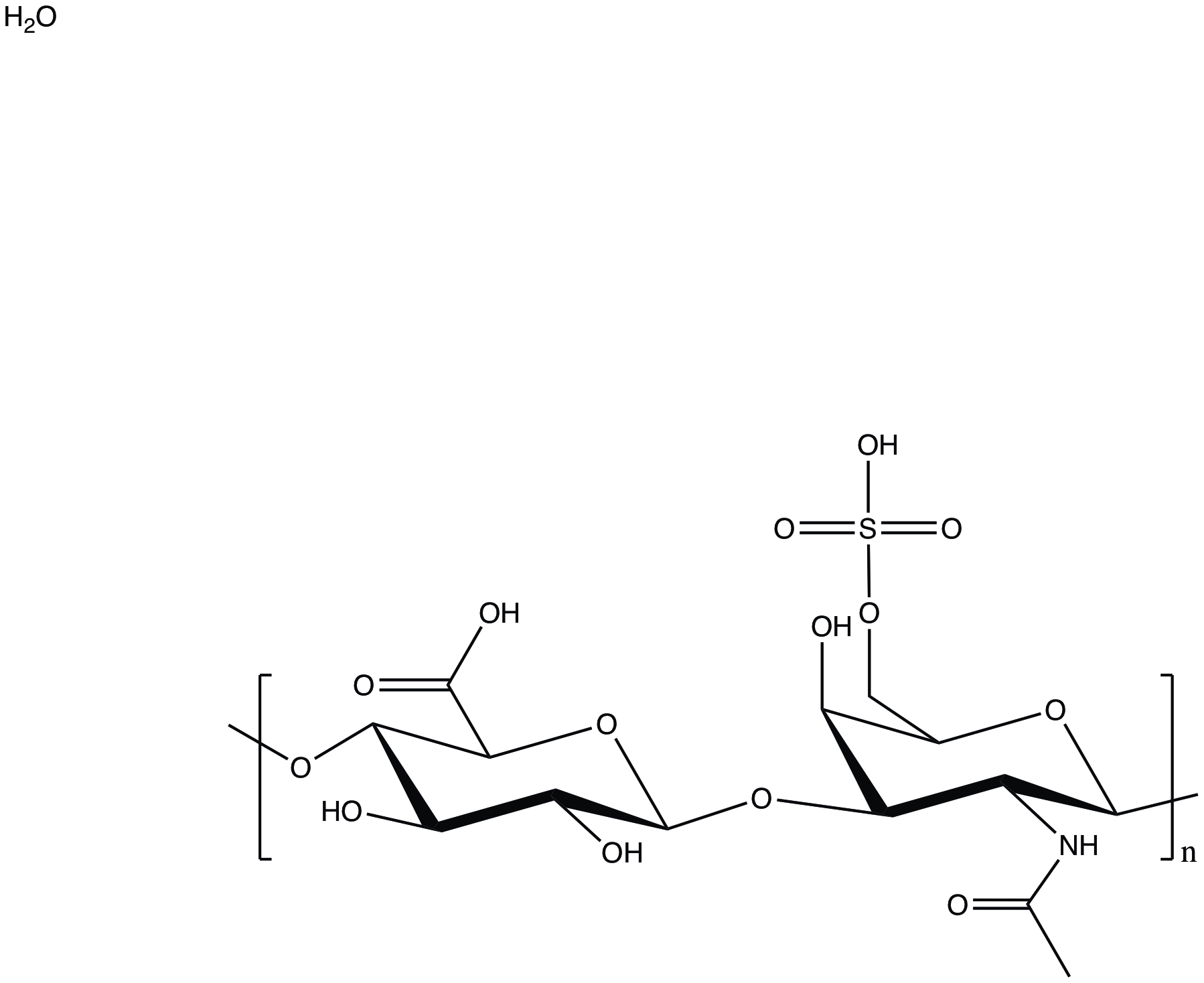 | 1.5 - 2[4] | 0.013 |  | - |

**References**

1. Zhang Y, Go EP, Jiang H, Desaire H. A novel mass spectrometric method to distinguish isobaric monosaccharides that are phosphorylated or sulfated using ion-pairing reagents. J Am Soc Mass Spectrom. 2005;16(11):1827-39.
2. Park W, Na K. Dermatan sulfate as a stabilizer for protein stability in poly(lactide-co-glycolide) depot. Biotechnology and Bioprocess Engineering. 2009;14(5):668-74.
3. Liao YH, Jones SA, Forbes B, Martin GP, Brown MB. Hyaluronan: pharmaceutical characterization and drug delivery. Drug Deliv. 2005;12(6):327-42.
4. Chandran PL, Horkay F. Aggrecan, an unusual polyelectrolyte: review of solution behavior and physiological implications. Acta Biomater. 2012;8(1):3-12.
